# Acute cardiac overload does not induce cardiac or skeletal expression of fibroblast growth factor 23 in rats

**DOI:** 10.1097/XCE.0000000000000249

**Published:** 2021-02-14

**Authors:** Abul Fajol, Hirotaka Komaba, Chigusa Ishioka, Takehiko Wada, Masafumi Fukagawa

**Affiliations:** a Division of Nephrology, Endocrinology and Metabolism; b Interactive Translational Research Center for Kidney Diseases, Tokai University School of Medicine; c The Institute of Medical Sciences, Tokai University, Isehara, Japan

**Keywords:** fibroblast growth factor 23, heart failure, volume overload

## Abstract

Elevated fibroblast growth factor 23 (FGF23) is associated with cardiovascular events, particularly heart failure. Although FGF23 has been reported to induce cardiac hypertrophy, recent studies demonstrated that cardiac hypertrophy and myocardial infarction induce FGF23 production by cardiomyocytes. We aimed to explore whether acute cardiac overload increases cardiac and skeletal *FGF23* expression and circulating FGF23 levels.

**Methods:**

We administered 30 μL/g bodyweight of isotonic saline intraperitoneally in rats to induce acute cardiac overload. We measured serum FGF23 levels and other parameters of mineral metabolism at 2, 6, and 24 h after saline or sham injection. We also analyzed gene expression in the heart, calvarium, femur, and kidney at 2 and 24 h after injection.

**Results:**

Acute saline injection induced cardiac overload as evidenced by a significant upregulation of brain natriuretic peptide along with a trend towards increased expression of atrial natriuretic peptide and mild hyponatremia. However, there were no changes in serum FGF23 levels or *FGF23* expression in the heart, calvarium, or femur.

**Conclusions:**

Acute cardiac overload by saline injection in rats did neither induce *FGF23* expression in the heart or bone nor did it increase serum FGF23 levels. These findings suggest that more severe or long-term cardiac damage is required for induction of *FGF23* expression.

## Introduction

Fibroblast growth factor 23 (FGF23) is a bone-derived hormone that plays a major role in the regulation of phosphate and vitamin D metabolism. FGF23 primarily acts on the kidneys to induce urinary phosphate excretion by downregulating the sodium-dependent phosphate cotransporters Napi2a and Napi2c and suppress 1,25-dihydroxyvitamin D [1,25(OH)_2_D] synthesis by altering the vitamin D-metabolizing enzymes CYP27b1 and CYP24a1 [1–3]. These functions of FGF23 are mediated by binding to FGF receptor 1 (FGFR1) and the transmembrane protein Klotho, which forms a specific receptor complex for FGF23 [4,5].

Importantly, recent observational studies have shown strong associations between elevated FGF23 and greater risk of cardiovascular events, particularly congestive heart failure, in patients with chronic kidney disease [6–8], hemodialysis patients [9,10], and individuals with apparently normal kidney function [11–15]. Furthermore, elevated FGF23 has been linked to volume overload, a common manifestation of heart failure [16–18].

As a potential mechanism of these observations, FGF23 is reported to induce cardiac hypertrophy via Klotho-independent signaling through the FGFR4 [19,20]. In addition, FGF23 increases renal sodium reabsorption by upregulating the Na^+^:Cl^−^ cotransporter in the distal tubules via a Klotho-dependent pathway, leading to volume expansion, hypertension, and cardiac hypertrophy [21]. Interestingly, recent studies demonstrated an opposite directional relationship—injured cardiomyocyte could produce substantial amounts of FGF23 after myocardial infarction [22] or during cardiac hypertrophy [23]. However, it remains to be determined whether acute volume expansion, which induces hemodynamic congestion and cardiac overload, stimulates FGF23 production by cardiomyocytes.

Here, we report our investigations into the impact of volume expansion by acute saline injection on cardiac and skeletal expressions of FGF23 as well as circulating FGF23 levels in rats.

## Materials and methods

### Animals

Five-week-old male Sprague–Dawley rats were purchased from CLEA Japan (Tokyo, Japan). The rats were housed under standard environmental conditions (23 ± 2°C, 55 ± 10% humidity, 12:12 h light-dark cycle) and had free access to water and standard rodent chow. At the age of 6 weeks, 30 μL/g bodyweight of isotonic saline was administered intraperitoneally to induce volume overload. Control rats received a sham injection (needle insertion only). Rats were then placed in metabolic cages for 24-h urine collection and were killed for further examination. A subset of rats was killed 2 h after saline or sham injection. All animal experiments were performed in accordance with institutional guidelines and were approved by the Institutional Animal Care and Use Committee at Tokai University.

### Blood pressure

We measured blood pressure at 2, 6, and 24 h after saline or sham injection using a computerized tail-cuff system (MK-2000; Muromachi Kikai, Tokyo, Japan). Before recording blood pressure, rats were trained to familiarize themselves with the measurement equipment. Blood pressure values were recorded five times and the results were averaged for each rat.

### Serum and urine biochemistries

Blood was collected from the tail vein or heart at 2, 6, and 24 h after saline or sham injection. Blood urea nitrogen (BUN), serum creatinine, sodium, calcium, and phosphorus, and urinary sodium levels were measured using standard methods. Serum parathyroid hormone (PTH) levels were measured using a rat intact PTH ELISA (Immutopics, San Clemente, California, USA). Serum FGF23 levels were measured using an intact FGF23 ELISA, which exclusively measures the full-length protein (Kainos Laboratories, Tokyo, Japan). Serum 1,25(OH)_2_D levels were measured using a radioimmunoassay (Immunodiagnostics Systems, Fountain Hills, Arizona, USA).

### Gene expression

Total RNA was isolated from the femur, calvarium, heart, and kidney using TRIzol reagent (Thermo Fisher Scientific, Waltham, Massachusetts, USA) according to the manufacturer’s instruction. For isolation from femurs, epiphyses were cutoff, bone marrow was removed by centrifugation, and bone tissue was homogenized using a Shake Master NEO (BioMedical Sciences, Tokyo, Japan) together with TRIzol reagent and 5-mm stainless beads. cDNA synthesis was performed using 0.5 µg RNA and SuperScript IV VILO Master Mix with ezDNase Enzyme (Thermo Fisher Scientific). Quantitative real-time PCR was performed on the StepOnePlus System (Thermo Fisher Scientific) using the TaqMan One-Step RT-PCR Master Mix Reagents kit (Thermo Fisher Scientific). Glyceraldehyde-3-phosphate dehydrogenase served as the reference gene to normalize expression.

### Statistical analysis

Data are represented as mean ± SD. Differences were evaluated using *t*-tests or mixed-effects models, as appropriate. *P* < 0.05 was considered statistically significant. All analyses were performed using IBM SPSS Statistics 24 (IBM, Tokyo, Japan).

## Results

**Fig. 1:**
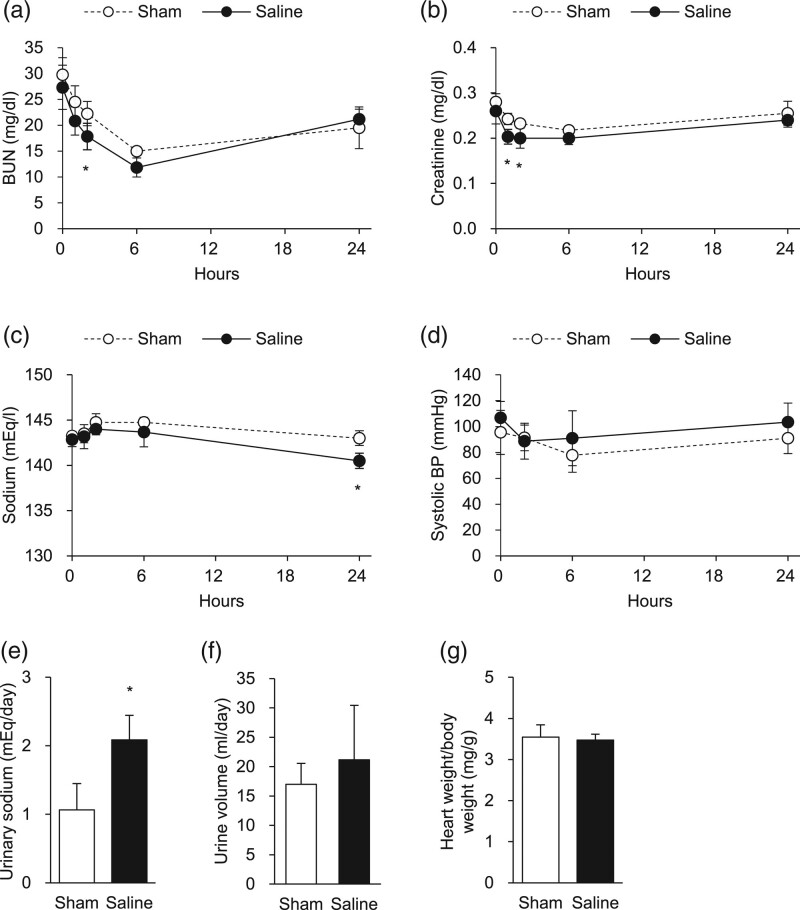
Cardiorenal effects of acute saline injection. (a–d) BUN (a), serum creatinine (b), sodium (c), systolic blood pressure (d) in sham rats (*n* = 4), and saline-injected rats (*n* = 6). (e, f) 24-h urine volume (e) and urinary sodium excretion (f) in sham rats (*n* = 4) and saline-injected rats (*n* = 6). (g) Heart weight/total body weight at 24 h after sham injection (*n* = 4) or saline injection (*n* = 6). Data are shown as mean ± SD. *P < 0.05 versus sham rats (*t*-tests or mixed-effects models). BUN, blood urea nitrogen.

Compared to sham-injected rats, saline-injected rats showed a transient and small decrease in BUN and serum creatinine (Fig. 1a and b), presumably as a result of dilution. The saline-injected rats also showed mild hyponatremia, a hallmark of heart failure, at 24 h after injection (Fig. 1c). These rats had higher systolic blood pressure (Fig. 1d) and diastolic blood pressure (data not shown) at 6 and 24 h, although the difference was not significant. As expected, 24-h urinary sodium excretion was markedly increased in saline-injected rats (Fig. 1e). The increase in urinary sodium excretion was virtually the same as the dose of sodium administered, suggesting recovery from volume expansion at 24 h. Urine volume also tended to increase in saline-injected rats (Fig. 1f), with a difference that was not significant but corresponded to the volume of saline administered.

**Fig. 2:**
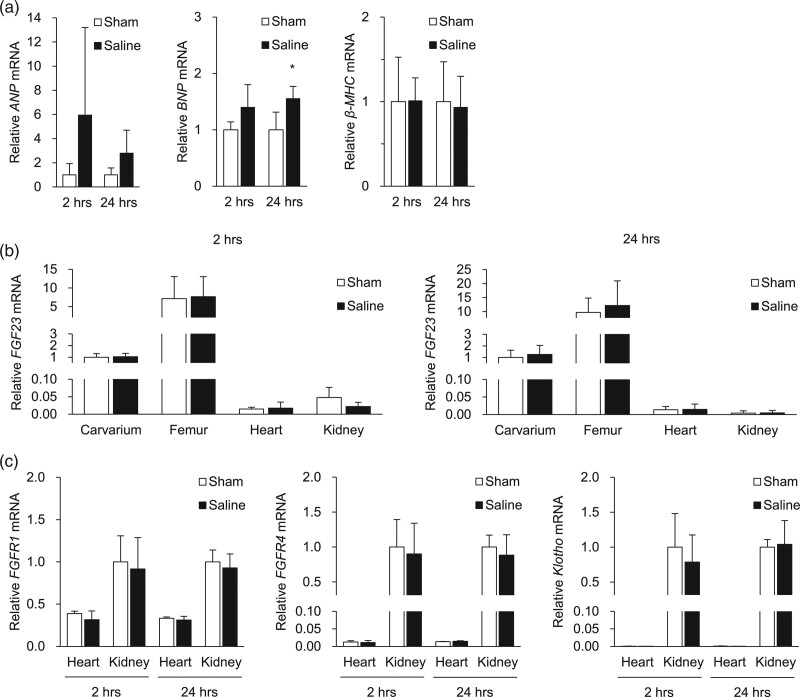
Effects of acute saline injection on gene expressions in the heart, bone, and kidney. (a) Quantitative real-time PCR of *ANP*, *BNP*, and *β-MHC* in the heart at 2 and 24 h after sham injection (*n* = 4) or saline injection (*n* = 6). (b) Quantitative real-time PCR of *FGF23* in the calvarium, femur, heart, and kidney at 2 and 24 h after sham injection (*n* = 4) or saline injection (*n* = 6). (c) Quantitative real-time PCR of *FGFR1*, *FGFR4*, and *Klotho* in the heart and kidney at 2 and 24 h after sham injection (*n* = 4) or saline injection (*n* = 6). Data are shown as mean ± SD. *P < 0.05 versus sham rats (*t*-tests). ANP, atrial natriuretic peptide; BNP, brain natriuretic peptide; β-MHC, β-myosin heavy chain; FGFR 1/4, fibroblast growth factor receptor 1/4; FGF23, fibroblast growth factor 23.

Next, we investigated the hearts obtained from the saline- and sham-injected rats. While the gross appearance and the ratio of heart weight standardized to total body weight did not differ between saline- and sham-injected rats (Fig. 1g), saline injection led to increased expression of *brain natriuretic peptide* (*BNP*) and a trend towards increased expression of *atrial natriuretic peptide* (*ANP*) (*P* = 0.07) (Fig. 2a), suggesting cardiac stress in response to volume overload. Expression of *β-myosin heavy chain* (*β-MHC*) was comparable between saline- and sham-injected rats, suggesting that the transient volume expansion after saline injection did not activate hypertrophic gene programs.

**Fig. 3:**
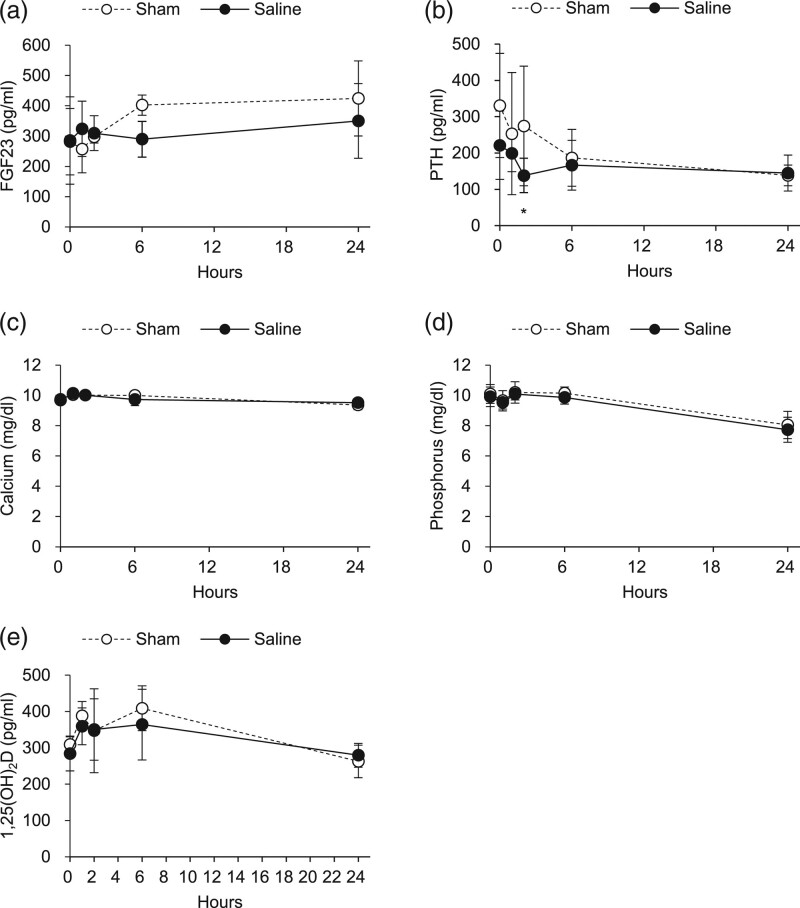
Effects of acute saline injection on parameters of mineral metabolism. Serum FGF23 (a), PTH (b), calcium (c), phosphorus (d), and 1,25(OH)_2_D (e) in sham rats (*n* = 4) and saline-injected rats (*n* = 6). Data are shown as mean ± SD. *P < 0.05 versus sham rats (mixed-effects models). FGF23, fibroblast growth factor 23; PTH, parathyroid hormone; 1,25(OH)_2_D, 1,25-dihydroxyvitamin D.

To explore whether cardiac overload by saline injection affects FGF23 synthesis by cardiomyocytes or osteoblasts/osteocytes, we examined gene expression in the heart and bone. We found no changes in *FGF23* expression in the heart, calvarium, or femur after saline injection (Fig. 2b). Recent studies reported weak but significant expression of *FGF23* in the kidney [24], but there was no difference in renal expression of *FGF23* between saline- and sham-injected rats. It is reported that FGF23 induces cardiac hypertrophy by Klotho-independent binding to FGFR4 [20]. However, the expression of *FGFR4* in the heart was much lower than that in the kidney, and these expressions did not change by saline injection (Fig. 2c). As expected, the expression of *Klotho* was abundant in the kidney but was almost undetectable in the heart. Overall, there was no significant difference in serum FGF23 levels between saline- and sham-injected rats (Fig. 3a), although serum FGF23 levels tended to decrease at 6 h after saline injection (*P* = 0.07). There was a transient decrease in serum intact PTH levels at 2 h after saline injection (Fig. 3b), which is the same time point when BUN and serum creatinine decreased (Fig. 1a and b). Serum calcium, phosphorus, and 1,25(OH)_2_D levels were comparable between saline- and sham-injected rats (Fig. 3c–e). We also measured the renal expression of genes involved in mineral metabolism, but there was no significant difference in expression of *Napi2a*, *Cyp27b1*, or *Cyp24a1* between saline- and sham-injected rats (data not shown).

## Discussion

Elevated FGF23 has emerged as having a strong association with cardiovascular events, cardiac hypertrophy, congestive heart failure, and volume overload [6–18]. Recent investigations demonstrated that cardiomyocytes are the source of FGF23 in animal models of myocardial infarction [22] and cardiac hypertrophy induced by transverse aortic constriction or cardiomyocyte-specific activation of calcineurin A [23]. Based on these reports, we hypothesized that acute cardiac volume overload stimulates FGF23 production by cardiomyocytes. To address this, we injected isotonic saline in rats, which produced a significant upregulation of *BNP* along with a trend towards increased expression of *ANP* and mild hyponatremia. However, there were no changes in serum FGF23 levels or *FGF23* expression in the heart, calvarium, or femur.

Our findings contrast with previous observations of increased serum FGF23 levels and increased cardiac expression of *FGF23* in rodents with myocardial infarction [22] and cardiac hypertrophy [23]. The major difference between those studies and ours is the etiology and severity of heart disease. While we were able to induce a significant upregulation of cardiac *BNP* by saline injection, the compensatory increase in urinary sodium excretion appeared to limit the volume expansion and thereby the pathological impact on cardiomyocytes, as suggested by the lack of changes in cardiac *MHC*. Thus, our findings that transient volume expansion by saline injection did not induce cardiac *FGF23* expression along with previous results in models of myocardial infarction [22] and more pronounced cardiac hypertrophy [23] suggest that induction of cardiac *FGF23* expression requires pathological processes in the heart such as hypertrophy and fibrosis. This possibility is further supported by recent human studies showing that circulating FGF23 levels were associated with the severity of heart failure [18], but these levels did not change after acute injection of isotonic saline in patients with arterial hypertension [25]. Future research should determine whether chronic volume expansion stimulates cardiac *FGF23* expression and whether this process requires hypertrophic growth of cardiomyocytes.

The synthesis of FGF23 by osteoblasts/osteocytes is mainly regulated by phosphate [26], calcium [27], 1,25(OH)_2_D [28], and PTH [29], but recent investigations revealed that aldosterone is also involved in this regulation [30]. Aldosterone acts on osteoblasts through the mineralocorticoid receptor to stimulate FGF23 production. It is unknown whether aldosterone also stimulates FGF23 synthesis in cardiomyocytes; but if so, there is a possibility that decreased aldosterone in response to volume expansion might have offset the effects of cardiac overload on cardiomyocyte expression of *FGF23*. Additional studies are required to address this possibility.

In the present study, we observed consistent reductions in BUN, serum creatinine, and intact PTH levels at 2 h after intraperitoneal saline injection, which could be explained by a dilution effect. However, no such reduction was observed for serum FGF23 levels at this time point, raising the possibility that volume expansion might have rapidly increased FGF23 secretion by bone cells in which a large amount of FGF23 is stored. However, this possibility is unlikely because serum FGF23 levels tended to decrease at 6 h after saline injection. Furthermore, the expression of *FGF23* in the bone did not change at 2 or 24 h after saline injection. Thus, we conclude that volume expansion by saline injection does not induce FGF23 production by bone cells.

Interestingly, our findings that cardiac overload did not enhance skeletal expression of *FGF23* are consistent with the study of cardiac hypertrophy [22], but not with another study showing that myocardial infarction induced *FGF23* expression in the bone as well as the heart [23]. These apparently conflicting results could be reconciled if we assume that injured cardiomyocytes after myocardial infarction release a factor that enhances *FGF23* expression in bone cells. Of note, a recent study has demonstrated that during acute kidney injury, kidney-derived glycerol-3-phosphate, a downstream product of glycolysis, acts on bone cells to stimulate FGF23 production [31]. Future research should determine whether glycerol-3-phosphate or other factors derived from injured cardiomyocytes are involved in the enhanced *FGF23* expression in bone cells after myocardial infarction.

### Conclusion

We demonstrated that acute cardiac overload by saline injection in rats did neither induce *FGF23* expression in the heart or bone nor did it increase serum FGF23 levels. These findings, together with previous results in models of myocardial infarction and cardiac hypertrophy, suggest that more severe or long-term cardiac damage is required for induction of *FGF23* expression. Additional studies are required to determine this possibility.

## Acknowledgements

A.F. is a JSPS International Research Fellow. We thank Ms. Sachie Tanaka and Mr. Shuho Hori, the Support Center for Medical Research and Education, Tokai University.

## Conflicts of interest

H.K. has received honoraria, consulting fees, or grant support from Bayer Yakuhin, Chugai Pharmaceutical, Japan Tobacco, Kyowa Kirin, Novartis, and Ono Pharmaceutical. T.W. has received honoraria, consulting fees, or grant support from Chugai Pharmaceutical, Daiichi Sankyo, Kyowa Kirin, and Otsuka Pharmaceutical. M.F. has received honoraria, consulting fees, or grant support from Astellas Pharma, Bayer Yakuhin, Kissei Pharmaceutical, Kyowa Kirin, Ono Pharmaceutical, and Torii Pharmaceutical. For the remaining authors, there are no conflicts of interest.
